# Decrease of laminin-511 in the basement membrane due to photoaging reduces epidermal stem/progenitor cells

**DOI:** 10.1038/s41598-020-69558-y

**Published:** 2020-07-28

**Authors:** Shunsuke Iriyama, Masahito Yasuda, Saori Nishikawa, Eisuke Takai, Junichi Hosoi, Satoshi Amano

**Affiliations:** 1 Shiseido Global Innovation Center, 1-2-11 Takashima, Nishi-ku, Yokohama, 220-0011 Japan; 2 Department of Dermatology, Gunma University Graduate School of Medicine, 3-39-22 Showa-Machi, Maebashi, Gunma, 371-8511 Japan

**Keywords:** Stem cells, Health care, Molecular medicine

## Abstract

Daily sunlight exposure damages the epidermal basement membrane (BM) and disrupts epidermal homeostasis. Inter-follicular epidermal stem cells (IFE-SCs) regulate epidermal proliferation and differentiation, which supports epidermal homeostasis. Here, we examine how photoaging affects the function of IFE-SCs and we identify key components in their cellular environment (niche). We found that sun-exposed skin showed a decrease of MCSP-positive and β1-integrin-positive cells concomitantly with a decrease of laminin-511 at the dermal–epidermal junction (DEJ), as compared with sun-protected skin. Higher levels of laminin-511 were associated with not only increased efficiency of colony formation, but also higher expression levels of MCSP as well as other stem cell markers such as Lrig1, ITGB1, CD44, CD46, DLL1, and K15 in keratinocytes from skin of 12- to 62-year-old subjects. UVB exposure to cultured human skin impaired laminin-511 integrity at the dermal–epidermal junction and reduced MCSP-positive basal epidermal cells as well as K15-positive cells. Combined treatment with matrix metalloproteinase and heparanase inhibitors protected the integrity of laminin-511 and inhibited the reduction of MCSP-positive cells and K15-positive cells. These results suggest that photoaging may reduce the levels of MCSP-positive and K15-positive epidermal stem/progenitor cells in the epidermis via loss of laminin-511 at the dermal–epidermal junction.

## Introduction

The skin is a multilayered organ that protects the organism against environmental stressors. The outermost layer of the skin is the epidermis, which has a high turnover rate owing to the continuous shedding (desquamation) of the uppermost cornified cells. This is a part of the process of forming the water-impermeable barrier, the stratum corneum. In human skin tissue, both the epidermal cell turnover rate and barrier function are impaired with aging. In skin tissue, which has a high cell turnover, the role of resident stem cells is crucial for ensuring equilibrium between cell loss and cell division, i.e., for maintaining homeostasis. Stem cells are instrumental in epidermal renewal, regeneration, and repair, and the integrity of a mammalian epidermis requires the proliferation of stem cells and the differentiation of their progeny^1^. Multiple pools of stem cells are located in different epidermal regions, including the permanent portion of the hair follicle (the bulge), the interfollicular epidermis, and the sebaceous glands^2^. Interfollicular epidermal stem cells (IFE-SCs) play an especially important role in epidermal homeostasis under normal conditions^3^. These cells express high levels of α6 and β1 integrins^4,5^, but lower levels of transferrin receptor CD71^6^. They also express Delta 1^7^, MCSP (melanoma-associated chondroitin sulphate proteoglycan)^8^, LRIG1 (Leu-rich repeats and immunoglobulin-like domains 1)^9^, p75 NGF receptor CD271^10^, and keratin-15^11^. It has been reported that MCSP- and CD271-expressing cells decrease in number as a person ages^12,13^. However, it is not known whether or not photoaging also causes alterations of the IFE-SCs population.

Stem cell functions, such as self-renewal, migration, and differentiation, are regulated by the cellular environment or niche, which is comprised of surrounding cells, the extracellular matrix (ECM), and soluble factors (growth factors, cytokines and chemokines)^14^. In the epidermis, the basement membrane (BM) has an important role in influencing the behavior of stem cells^15^. The epidermal BM at the dermal–epidermal junction (DEJ) is a ubiquitous sheet-like polymeric structure that binds the dermis and the epidermis of the skin together. It is mainly composed of type IV and type VII collagens, several laminins (such as 332, 521 and 511), nidogen, and perlecan^16^. The epidermal BM is damaged in skin that is regularly exposed to sunlight^17^, since several injurious enzymes in the epidermis are activated by UV irradiation^18,19^. IFE-SCs are regulated by laminin-332 and laminin-511^15,20^, and the levels of these molecules are reduced with aging^20,21^. However, it is not clear whether sunlight exposure influences the age-dependent change of laminins in the skin.

Laminins have been implicated in the control of stem cell maintenance and progenitor cell differentiation in various adult tissues. Laminin-332 and laminin-511 are present at the BM in the hair follicle, and the precise ratio of laminin-332/laminin-511 is critical for maintaining stem cell homeostasis^15^. The subventricular zone of the lateral ventricle is a stem cell niche in the adult brain, containing a population of neural stem cells that are able to self-renew and differentiate^22^. Our previous study using a LAMA5-deficient mouse model showed that laminin α5 plays an important role in maintaining neural stem cells as a part of the stem cell niche in the subventricular zone^23^. In mammary glands, laminin-integrin interactions play an essential role in controlling the proliferative potential of mammary basal stem/progenitor cells^24^. Laminins also regulate stem cell maintenance and muscle development^25^. Laminin α5 in the stem cell niche affects stem cell proliferation and self-renewal, leading to muscle regeneration^26^.

We have shown that matrix metalloproteinases (MMPs) and heparanase are activated in UVB-irradiated human skin^27,28^, and also that UV-damaged BM structure is reconstituted in the presence of inhibitors of MMPs and heparanase, concomitantly with the induction of epidermal differentiation markers such as filaggrin, loricrin, and bleomycin hydrolase in the granular layer of the epidermis^28^. This leads to improved barrier function^29,30^. However, it is not known whether MMP and heparanase inhibitors promote the deposition of laminins at the DEJ in UVB-irradiated human skin.

Although the interaction between IFE-SCs and their environmental niche is critical for the maintenance of epidermal homeostasis, the roles of the IFE-SC niche components are not fully understood. Therefore, the aim of this work is to examine how photoaging affects the function of the IFE-SCs and to identify key components in the cellular environment (niche) by comparing sun-exposed skin and sun-protected skin of subjects at various ages. We also examined the role of niche components using organotypic human skin exposed to UV irradiation.

## Results

### MCSP-positive inter-follicular epidermal stem/progenitor cells were reduced with aging in sun-exposed human skin, compared with sun-protected skin

**Figure 1:**
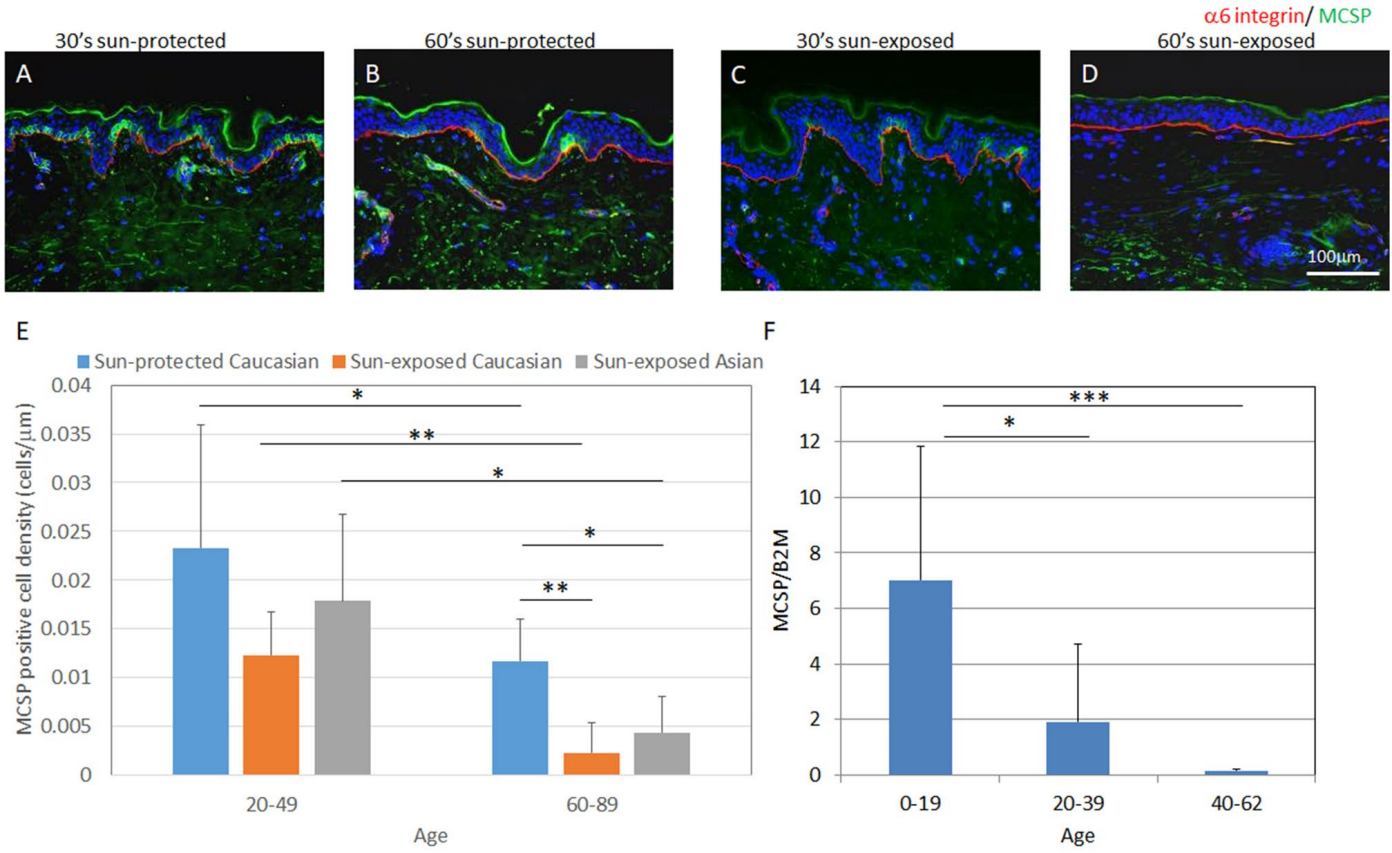
Age-dependent change of MCSP-positive cells in sun-protected and sun-exposed skin. Immunofluorescence staining of MCSP (green) and α6 integrin (red) in 30s sun-protected Caucasian skin (**A**), 60s sun-protected Caucasian skin (**B**), 30s sun-exposed Caucasian skin (**C**) and 60s sun-exposed Caucasian skin (**D**) [numbers indicate the age range (decade) of the skin donors]. MCSP-positive cell density was analyzed using WINROOF 2013 image analyzing software (Mitani, Fukui, Japan, https://www.mitani-visual.jp/products/image_analys_ismeasure-ment/winroof/) (**E**). mRNA expression level of MCSP was analyzed by qPCR (**F**). Bars: 100 μm. Data are expressed as mean ± SD of three independent experiments from each of 6 different donors in **E** and from each of 5 donors in **F**. *p < 0.05., ***p < 0.001.

**Figure 2:**
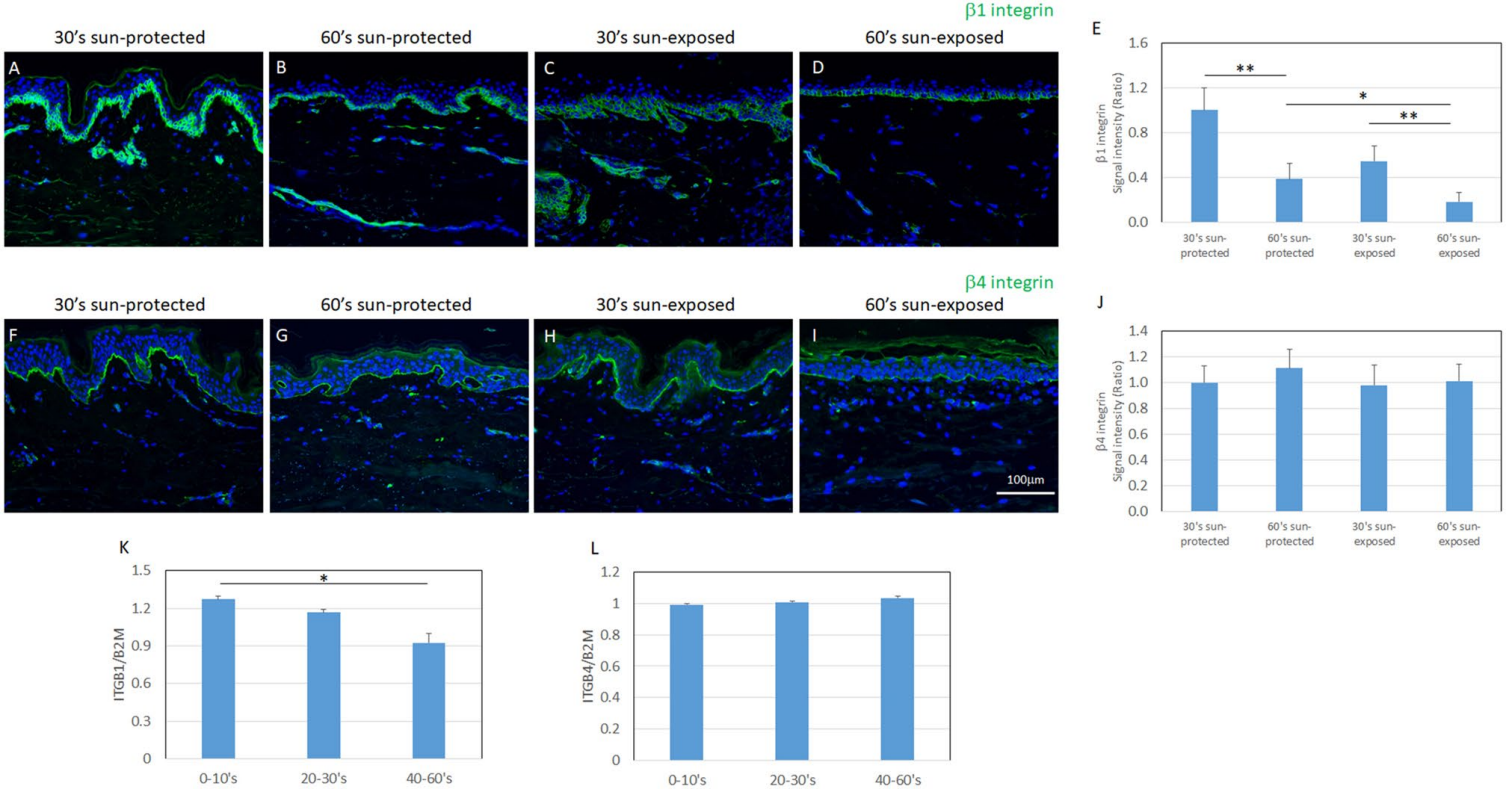
Age-dependent change of integrins. Immunofluorescence staining of β1 integrin (green) and α6 integrin (red) in 30s sun-protected Caucasian skin (**A**), 60s sun-protected Caucasian skin (**B**), 30s sun-exposed Caucasian skin (**C**) and 60s sun-exposed Caucasian skin (**D**) [numbers indicate the age range (decade) of the skin donors]. Immunofluorescence staining of β4 integrin (green) and α6 integrin (red) in 30s sun-protected Caucasian skin (**F**), 60s sun-protected Caucasian skin (**G**), 30s sun-exposed Caucasian skin (**H**) and 60s sun-exposed Caucasian skin (**I**). β1 integrin’s signal intensity (**E**) and β4 integrin’s signal intensity (**J**) were analyzed using WINROOF 2013 image analyzing software (Mitani, Fukui, Japan, https://www.mitani-visual.jp/products/image_analys_ismeasurement/winroof/). mRNA expression levels of ITGB1 (**K**) and ITGB4 (**L**) were analyzed by qPCR. Data are expressed as mean ± SD from each of 5 donors in **I** and **J**. *p < 0.05, **p < 0.01. Bars: 100 μm.

In human skin IFE-SCs are known to highly express MCSP^8^, β1 integrin^4,5^ and keratin-15 and to be reduced with aging^12^. To investigate the age-dependent changes of MCSP-positive cells, we performed immunofluorescence staining of MCSP in sun-exposed and sun-protected skin samples. Age-dependent reduction of MCSP-positive cells was more pronounced in sun-exposed skin than in sun-protected skin (Fig. 1A–E). In addition, the gene expression level of MCSP in cultured keratinocytes from sun-protected skin of various ages decreased with aging (Fig. 1F). Similarly, the signal intensity of β1 integrin decreased earlier in sun-exposed skin than in sun-protected skin as compared with β4 integrin’s intensity (Fig. 2A–J). The gene expression level of Itgb1 decreased with aging whereas Itgb4 showed no change with aging (Fig. 2K, L). Furthermore, keratin-15-positive cells also decreased in number in sun-exposed skin (Fig.S1A–E), and the gene expression level of K15 was reduced with aging (Fig.S1F), although the location of K15-positive cells might be different from that of MCSP-positive cells. Thus, our findings indicate that the aging-related decrease of IFE-SCs occurs earlier in sun-exposed skin than in sun-protected skin.

### Laminin-511 was reduced age-dependently in sun-exposed skin

**Figure 3:**
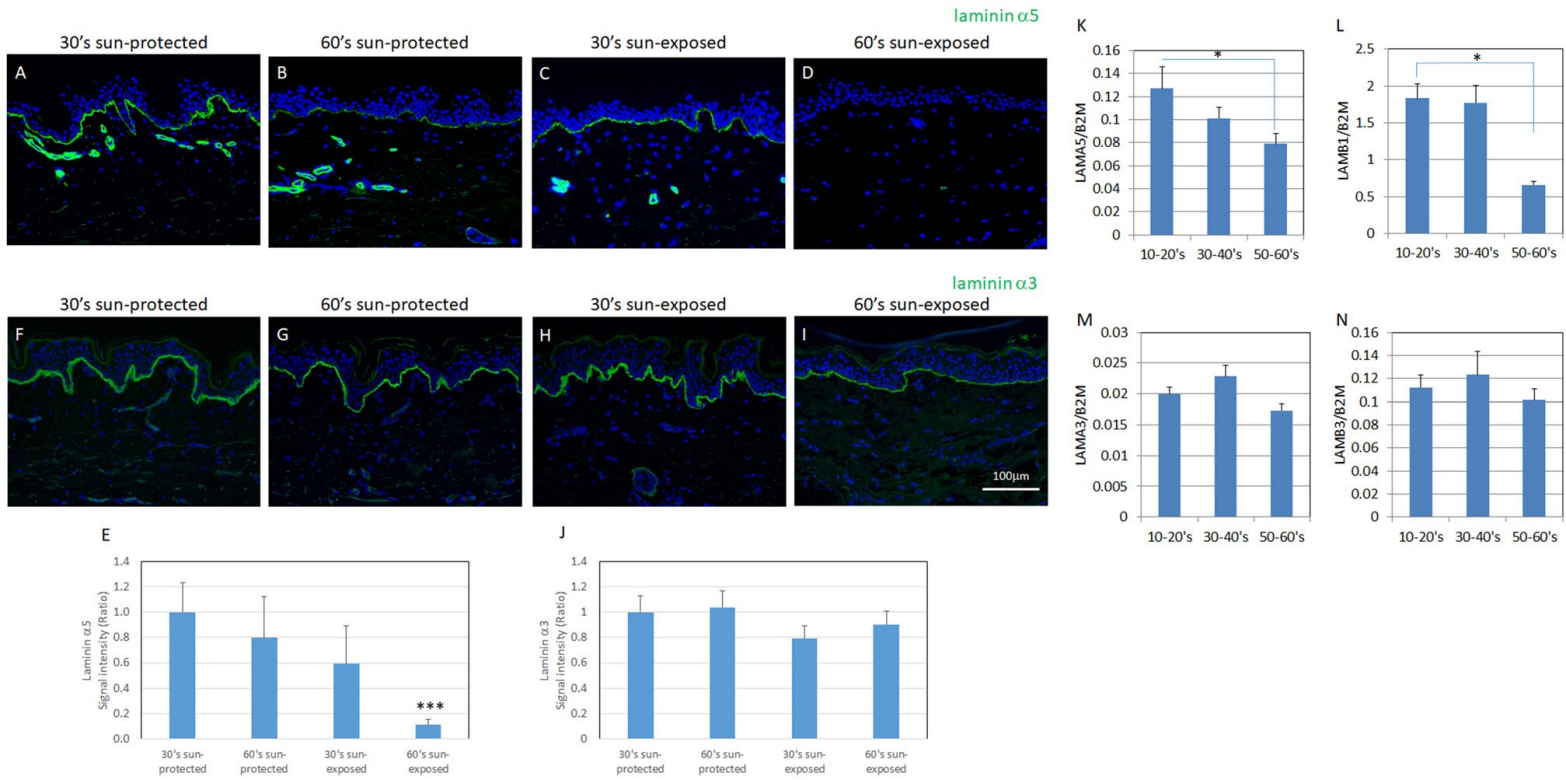
Age-dependent change of laminins. Immunofluorescence staining of laminin-α5 in 30s sun-protected Caucasian skin (**A**), 60s sun-protected Caucasian skin (**B**), 30s sun-exposed Caucasian skin (**C**) and 60s sun-exposed Caucasian skin (**D**) [numbers indicate the age range (decade) of the skin donors].Immunofluorescence staining of laminin-α3 in 30s sun-protected Caucasian skin (**F**), 60s sun-protected Caucasian skin (**G**), 30s sun-exposed Caucasian skin (**H**) and 60s sun-exposed Caucasian skin (**I**). Laminin α5′s signal intensity (**E**) and laminin α3′s signal intensity (**J**) were analyzed using WINROOF 2013 image analyzing software (Mitani, Fukui, Japan, https://www.mitani-visual.jp/products/image_analys_ismeasurement/winroof/). mRNA expression levels of LAMA5 (**K**), LAMB1 (**L**), LAMA3 (**M**) and LAMB3 (**N**) were analyzed by qPCR. Bars: 100 μm. Data are expressed as mean ± SD of three independent experiments from each of 5 donors in **I**–**L**. *p < 0.05.

In the epidermis, undifferentiated stem/progenitor cells residing at the basal layer bind to the basement membrane via integrin-laminin interaction. We next investigated the integrin-laminin interaction of IFE-SCs by immunofluorescence staining of laminin α5 and laminin α3. We found that laminin α5 was clearly reduced in sun-exposed skin (Fig. 3A–E), whereas laminin α3 was not (Fig. 3F–J). We examined the gene expression levels of LAMA5, LAMA3, LAMB3 and LAMB1 in cultured keratinocytes from sun-protected skin in subjects of various ages and compared them with the histological data. Consistent with the age-dependent reduction of laminin α5, we found that the expression levels of LAMA5 and LAMB1 were significantly reduced with aging (Fig. 3K–L). However, the expression levels of LAMA3 and LAMB3 did not change with aging (Fig. 3M, N). Thus, our findings indicate that laminin-511 was decreased more in sun-exposed skin compared to laminin-332. In addition, the gene expression levels of LAMA5 and LAMB1 were unchanged whereas the gene expression of MMP-9 was increased in UVB-irradiated cultured keratinocytes (Supplementary Fig. S2), suggesting that UVB irradiation might lead to the degradation of laminin-511.

### Epidermal stem cell population was maintained by laminin-511

**Figure 4:**
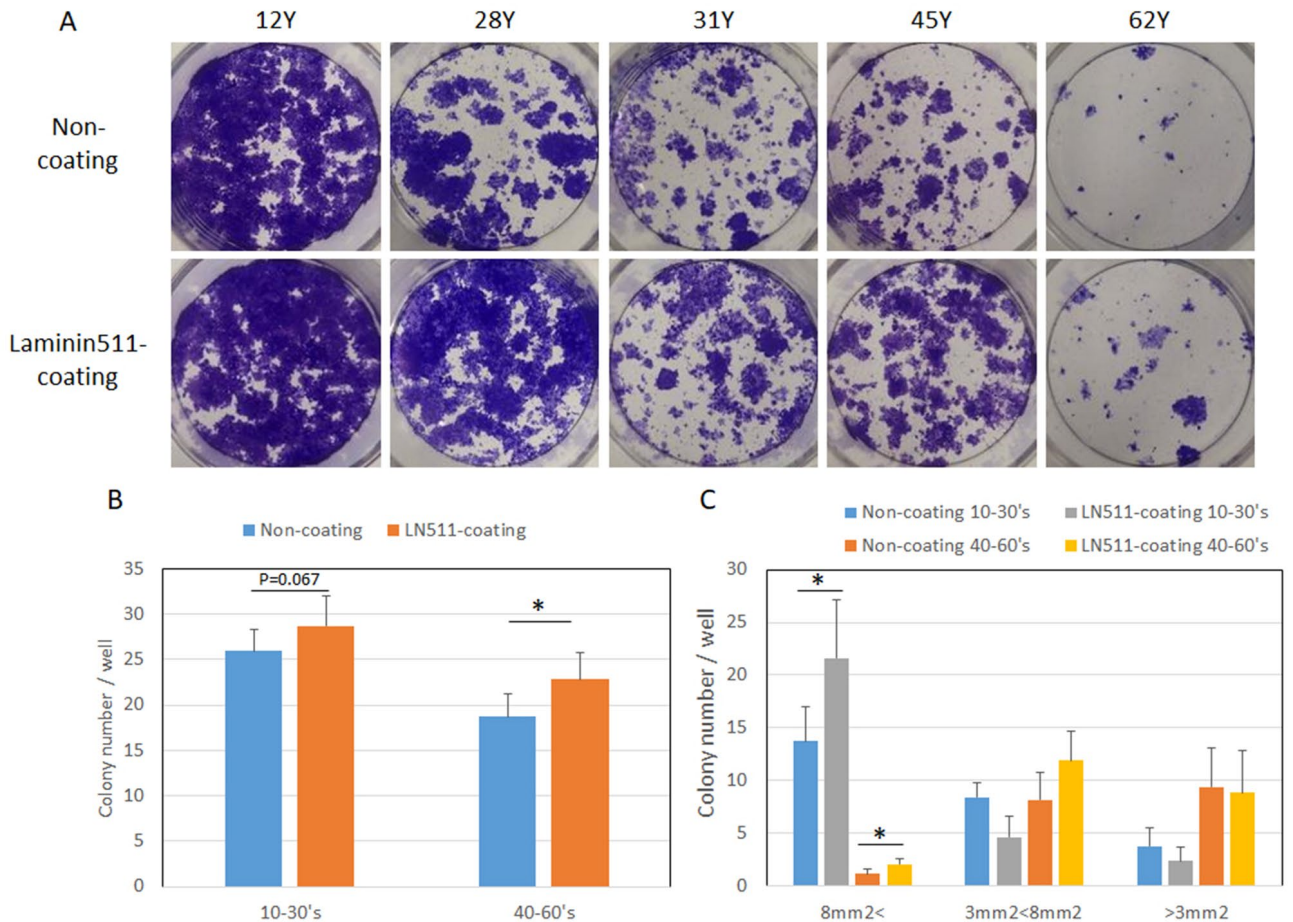
Epidermal stem/progenitor cell populations were maintained in the presence of laminin-511. Colony formation assay using human keratinocytes from donors of different ages after pre-culture on laminin-511-coated and non-coated plates (**A**). Colony number and size were analyzed using WINROOF 2013 (**B**, **C**). Data are expressed as the mean ± SD from each of 6 different donors in **B** and **C**. *p < 0.05.

**Figure 5:**
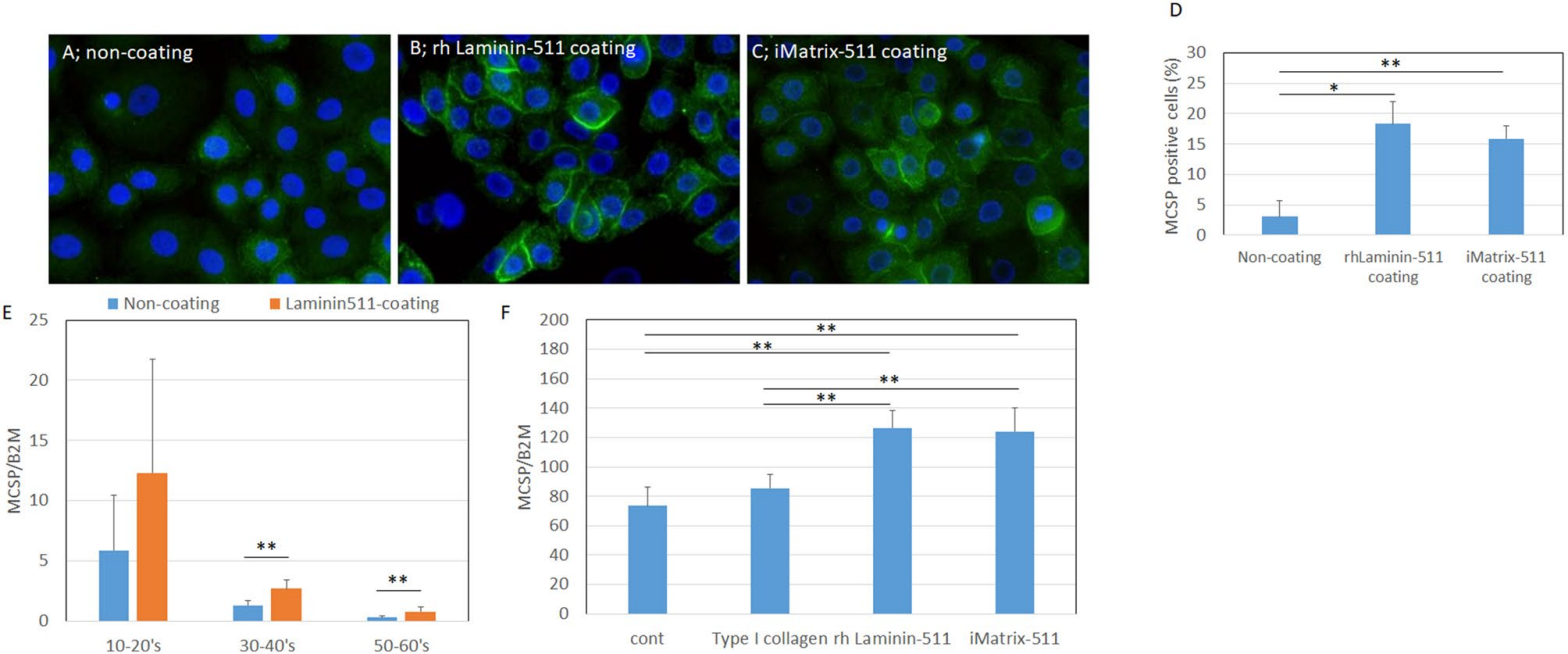
Epidermal stem cell markers were maintained in the presence of laminin-511. Immunofluorescence staining of MCSP in cultured neonatal keratinocytes on non-coated plates (**A**), and plates coated with recombinant human laminin-511 (**B**) or iMatrix-511 (**C**). Immunofluorescence was performed in three independent experiments, and average images are shown in **A**–**C**. MCSP-positive cell (%) was analyzed using WINROOF 2013 image analyzing software (Mitani, Fukui, Japan, https://www.mitani-visual.jp/products/image_analys_ismeasurement/winroof/) (**D**). The mRNA expression level of MCSP in cultured keratinocytes from donors of various ages was analyzed by qPCR (**E**). The mRNA expression levels of MCSP were analyzed by qPCR (**F**). Data are expressed as mean ± SD of three independent experiments from each of 5 donors in **E** and from 3 of the donors in **F**. Statistical analysis in **D** and **E** were performed using ANOVA with the Tukey–Kramer post hoc test: **p < 0.01. Bars: 100 μm.

Since decreased levels of laminin-511 and α6β1 integrin during aging in sun-exposed skin were associated with the reduction of MCSP-positive epidermal stem cells, we examined whether laminin-511 could reduce the loss of inter-follicular epidermal stem/progenitor cells by means of a colony formation assay on laminin-511-coated plates. Human keratinocytes from several aged donors showed higher colony numbers and greater colony sizes on the coated plates, compared with the controls (Fig. 4A–C). In addition, MCSP-positive cell populations (%) increased on plates coated with laminin-511 or iMatrix-511, which consists of E8 fragments of recombinant laminin-511 (Fig. 5A–D). Moreover, when human keratinocytes from donors of several ages were grown on laminin-511-coated plates, the mRNA level of MCSP increased (Fig. 5E). Laminin-511 and iMatrix-511 were better matrix proteins than type I collagen and non-coated controls in neonatal keratinocytes (Fig. 5F). The mRNA levels of other stem cell markers, Lrig1, ITGB1, CD44, CD46, DLL1, and K15 were also increased in neonatal keratinocytes cultured on type I collagen, laminin-511, or iMatrix-511, as compared to non-coated controls (Fig. S3). In addition, the mRNA levels of these stem cell markers MCSP, Lrig1, CD46, CD44, and DLL1 were increased on iMatrix-511 and were higher in keratinocytes from a 22-year-old donor than a 54-year-old donor (Supplementary Fig. S4). These results indicate that laminin-511 may play an important role in supporting epidermal stem/progenitor cell populations.

### Blocking loss of laminin-511 in UVB-exposed and UVB-unexposed organotypic human skin maintained MCSP-positive cell levels

**Figure 6:**
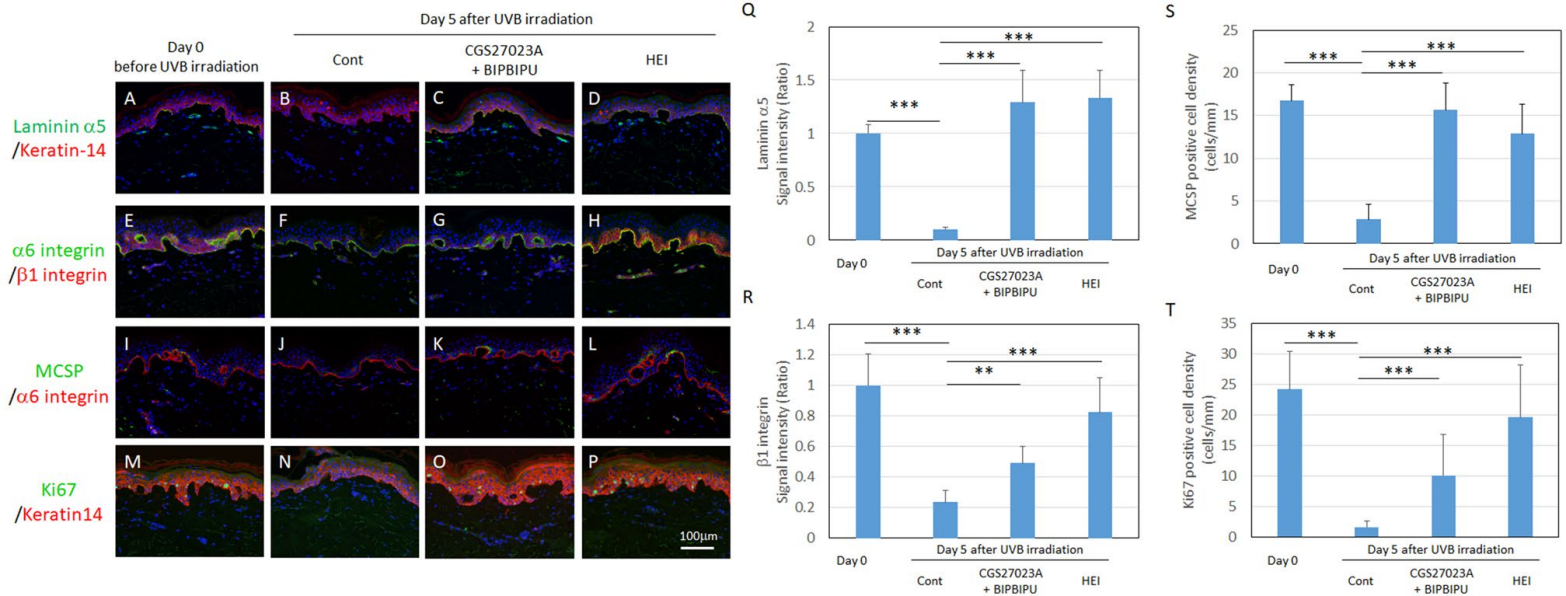
Epidermal stem/progenitor cells were increased in the presence of MMP inhibitor and heparanase inhibitor in the UVB-exposed OC model. Immune-fluorescence staining intensities of laminin-α5 (**A**–**D**), β1 integrin (**E**–**H**), MCSP (**I**–**L**) and Ki67 (**M**–**P**) were compared in un-cultured skin (**A**, **E**, **I**, **M**), and in UVB-exposed organotypic human skin without treatment (**B**, **F**, **J** and **N**), after treatment with MMP inhibitor CGS27023A and heparanase inhibitor BIPBIPU (**C**, **G**, **K** and **O**), and after treatment with bifunctional inhibitor HEI (**D**, **H**, **L** and **P**). Signal intensity (Ratio) of laminin-α5 (**Q**) and b1 integrin (**R**), and MCSP-positive cell density (**S**) and Ki67-positive cell density (**T**) were analyzed using WINROOF2013 image analyzing software (Mitani, Fukui, Japan, https://www.mitani-visual.jp/products/image_analys_ismeasurement/winroof/). Data are expressed as the mean ± SD of the three independent experiments from each of 4 donors in **Q**–**T** as shown in Table S2. ***p < 0.001. Bar, 100 μm.

We previously reported that UVB exposure induced and activated MMP-9 and heparanase in the epidermis, and damaged the structure of the basement membrane at the DEJ, leading to a reduction of proliferative keratinocytes and barrier disruption^18,19,30^. We also showed that MMP and heparanase inhibitors suppressed the degradation of the basement membrane and maintained proliferative keratinocytes and epidermal barrier function in UVB-exposed organotypic human skin^29,30^. Thus, we next performed ex vivo human skin cultures in the presence of MMP inhibitor CGS27027A, heparanase inhibitor BIPBIPU, and bifunctional inhibitor HEI after UVB exposure, in order to investigate the mechanisms underlying the effect of laminin-511 on epidermal stem/progenitor cells. Histological analysis showed that the UVB-induced decrease of laminin-α5 (Fig. 6A, B) was inhibited in presence of the MMP inhibitor and the heparanase inhibitor (Fig. 6B–D, Q), and immunofluorescence staining revealed that the densities of β1 integrin-, MCSP-, or K15-positive cells in the epidermis were restored (Fig. 6E–L, R, S and Fig. S5A–E). Further, Ki67-labeled proliferative cells in the basal layer of the interfollicular epidermis were substantially maintained by treatment with these inhibitors after UVB irradiation (Fig. 6M–P, T). Together, these results indicate that preventing the loss of laminin-511 may be essential for the maintenance of inter-follicular epidermal stem/progenitor cells in UVB-exposed organotypic human skin.

## Discussion

Stem cell exhaustion is a well-established characteristic of aging^31,32^, leading to reduced regenerative potential of tissues, including mouse forebrain^33^, bone^34^, and muscle fibers^35^. In skin, epidermal stem cells are also reduced with aging in the bulge region^36^, and inter-follicular epidermis^12,13^. Our findings here show that MCSP- and K15-positive epidermal cells are reduced with aging at an earlier stage in sun-exposed facial skin, as compared with sun-protected skin. IFE-SCs are known to generate daughter cells continuously to maintain the rete ridge height^12^, and our findings are consistent with a previous report showing that rete ridge height is more markedly reduced with aging in photoaged skin than in sun-protected skin^12,17, 37–40^.

Our findings also show that the reduction of MCSP-positive epidermal stem cells during aging was associated with a decreased level of laminin-511, but not with a change of laminin-332. Laminin-511 at the basement membrane is known to serve to maintain hair follicle stem cells^15^. Recently laminin-511 was reported to be a useful matrix to culture iPS/ES cells and keratinocytes without the need for a feeder layer^41–43^. β1 Integrin, a stem cell marker, interacts with laminin-511 at the basement membrane^44^ and asymmetric cell division in the epidermis is disrupted in β1 integrin KO mice, but not in β4 integrin KO mice^45^. Moreover, laminin-511 in the embryo and uterus is involved in the progression of implantation^46^. Although laminin-332 is also an important matrix component at the basement membrane, as it interacts with α6β4 integrin in the hemidesmosomes connecting keratinocytes to the underlying dermis^47^, it mainly functions in regulating epidermal differentiation^20^. Therefore, laminin-511 may play a more important role than laminin-332 in maintaining MCSP-positive epidermal stem/progenitor cells.

The location of human quiescent epidermal stem cells within the basal layer is still under debate, and two different hypotheses have been proposed. One is that cells with the highest expression of β1 integrin, MCSP and LRIG1 cluster at the top of the rete ridges and harbor the highest stemness of all basal cells^8,9,40^. The other is that cells with the highest expression of α6 integrin and keratin-15 are located at the bottom of the rete ridges and are relatively quiescent, displaying a higher clonogenic potential than the more proliferative and keratin-15-dimer-positive upper-rete ridge basal cells^6,11,48^. In this study, we found that both keratin-15-positive cells at the bottom of the rete ridges and MCSP-positive cells at top of the rete ridges were reduced with aging and by sun-exposure and maintained by protecting the degradation of laminin-511. Thus, these data suggest that laminin-511 may be involved in supporting basal cells by maintaining stem/progenitor cells, such as MCSP-positive cells and K15-positive cells.

We found that the aging-related decline of laminin-511 was greater in sun-exposed facial skin than in sun-protected skin. Laminin-511 was reported to decrease significantly with aging in sun-protected human skin^21^. Laminin-511 is known to be associated with perlecan networks in basement membrane^49^ by binding to heparan sulfate proteoglycan^50,51^. We previously reported that heparanase was activated in the epidermis of UVB-exposed skin, leading to degradation of heparan sulfate of perlecan in the basement membrane at the DEJ^29,40,52^. Furthermore, treatment with inhibitors of MMPs and heparinase not only protected the heparan sulfate staining but also blocked the loss of laminin-511 at the DEJ in organotypic human skin^29,30^. In addition, we found that UVB irradiation did not change the gene expression levels of LAMA5 and LAMB1 in cultured keratinocytes (Supplementary Fig. S2). All of these data suggest that laminin-511 in the epidermis may be destabilized with aging via the increased degradation of heparan sulfate chains due to the activation of heparanase.

UV exposure is known to be one of the factors that enhance skin aging. We have been studying the role of the basement membrane in the process of skin aging, especially the photoaging, which occurs in the sun (UV)-exposed skin. We reported that basement membranes at the DEJ were damaged and the lamina densa became disrupted and/or multilayered in sun-exposed skin. Recently, Liu et al. reported that the UV-induced degradation of the hemidesmosome component collagen XVII at the basement membrane in the DEJ might lead to hemidesmosome instability and stem cell competition, and basal keratinocytes highly expressing collagen XVII dominate as interfollicular epidermal stem cells in aged epidermis^53^. We found that the combined use of matrix metalloproteinase and heparanase inhibitors protected basement membrane integrity and promoted the stability of collagen XVII localization^54^, and our present results show that this treatment also inhibited the reduction of stem/progenitor cells. Therefore, protection of the basement membrane from damage may be one of the best methods of controlling the skin aging process.

## Materials and methods

### Subjects

Sun-exposed facial and sun-protected human abdominal skin samples were obtained from Biopredic International, Rennes, France. Helsinki principles were adhered to, and participants gave written, informed consent to provide samples for research. Some sun-exposed facial skin samples were obtained with the approval of the ethics committees at Gunma University and Shiseido. The experimental protocols were also approved by both ethics committees. The samples were from subjects in the age range of 20–80 years. The characteristics of the samples are summarized in Supplementary Table S1. Skin samples were fixed in cold acetone (AMeX procedure) and embedded in paraffin for immunohistochemical observation using specific antibodies for Figs. 1, 2, 3 and S1.

### Materials

Heparanase inhibitor 1-[4-(1H-benzoimidazol-2-yl)phenyl]-3-[4-(1H-benzoimidazol-2-yl)phenyl]urea (BIPBIPU), MMPs inhibitor *N*-hydroxy-2(R)-[[(4-methoxyphenyl)sulfonyl](3-picolyl)amino]-3-methylbutanamide hydrochloride (CGS27023A), and bifunctional inhibitor hydroxyethyl-imidazoridinone (HEI) were synthesized at Shiseido Co. Ltd. (Yokohama, Japan) according to the literature^52,55,56^.

### Organotypic human skin model

Fresh human abdominal skin (4 samples, from females in their 20s to 30s; the characteristics of the samples are summarized in Supplementary Table S2) was cut into 1.5 × 1.5 cm pieces, which were cultured in the presence or absence of 0.1 mg/mL HEI, BIPBIPU (10^−5^ M) or CGS27023A (10^−5^ M) after UVB irradiation (50 mJ/cm^2^), as described previously^30^. At 5 days after the start of culture, samples were fixed in cold acetone (AMeX procedure) and embedded in paraffin for immunohistochemical staining for Fig. 6 and S5.

### Immunohistochemistry

Each section was incubated overnight at 4 °C with a primary antibody together with guinea pig antiserum against cytokeratin-14 (this stains the epithelial compartment of skin). An Alexa Fluor 488–conjugated (green) secondary antibody directed towards the species of the primary antibody (1:200; Molecular Probes) and Alexa Fluor 594–conjugated (red) anti-guinea pig secondary antibody (1:200; Molecular Probes) were used to visualize primary antibodies, which targeted MCSP (MAB2029, Millipore, Darmstadt, Germany), α6 integrin (GOH3, Santa Cruz, CA), cytokeratin-15 (LHK15, Thermo Scientific, IL), β1 integrin (P5D2, Santa Cruz, CA), β4 integrin (MAB1964, Millipore, Darmstadt, Germany), laminin α5 (MABT39, Millipore, Darmstadt, Germany) and cytokeratin-14 (20R-CP002, Fitzgerald, MA). Sections were examined with an Olympus BX51 microscope (Olympus, Tokyo, Japan) and images were captured with a DP72 controller digital camera (Olympus). The staining intensity in 5 randomly selected microscopic fields was quantified by using WINROOF2013 image analyzing software (Mitani, Fukui, Japan, https://www.mitani-visual.jp/products/image_analys_ismeasurement/winroof/) as described previously^30^.

### Cell culture

Human normal epidermal keratinocytes obtained from donors of various ages (for details, see Supplementary Table S3) were cultured in Humedia-KG2 (KURABO, Osaka, Japan) in flasks coated with iMatrix-511 (Nippi, Tokyo, Japan) or recombinant human laminin-511 (BioLamina, Sundbyberg, Sweden). The flasks were incubated at 37 °C in a humidified atmosphere with 5% CO_2_. Keratinocytes (2.5 × 10^5^ cells) were seeded into 6-well plates coated or not coated with iMatrix-511 or recombinant human laminin-511, and incubated for 48 h. Cultured keratinocytes were used for qPCR analysis and immunofluorescence at passage 3. RNA was extracted from each sample for quantitative PCR analysis for Figs. 1, 2, 3, 5, S2, S3, S4, and keratinocytes were fixed with paraformaldehyde for MCSP immunofluorescence for Fig. 5.

### Quantitative real-time RT-PCR

Total epidermal RNA from cultured keratinocytes was isolated using a Qiagen Rneasy mini kit (Qiagen) and cDNA was synthesised using a SuperScript VILO cDNA Synthesis Kit (Thermo Fisher Scientific). Expression of MCSP, B2M, CK15, LAMA5, LAMB1, LAMA3, LAMB3, CD46, Lrig1, DLL1 and CD44 genes in the epidermis was analyzed by a quantitative PCR method using Platinum SYBR Green qPCR superMix-UDG (Invitrogen Japan, Tokyo, Japan), as described^28^. Primer sequences were as follows: B2M forward, 5′-GTGGGATCGAGACATGTAAGCA-3′, B2M reverse, 5′-CAATCCAAATGCGGCATCT-3′, MCSP forward, 5′-CACGGCTCTGACC-GACATAG-3′, MCSP reverse, 5′-CCCAGCCCTCTACGACAGT-3′, CD46 forward, 5′-CTTTGTA-GTCTCTGGCAAGATGC-3′, CD46 reverse, 5′-CGGG-TATAAACTTCAACTCTGTGC-3′, LRIG1 forward, 5′-CTTGACCTGGG-TTCTGGGTA-3′, LRIG1 reverse, 5′-GGCCAAAGGAACATTTGAAG-3′, DLL1 forward, 5′-TCCAAGGATATATGCCCCAA-3′, DLL1 reverse, 5′-GA-ACTCGGTTTCTCAGCA-GC-3′, CD44 forward, 5′-TTGCAGTCAA-CAGTCGAA-3′, CD44 reverse, 5′-T-TCTGACGACTCCTTGTTC-3′, LAMA5 forward, 5′-TGGCTGGATTATGTAC-TCGTGG-3′, LAMA5 reverse, 5′-CTG-TAGCACCTACTTCGTGGCA-3′, LAMB1 forward, 5′-TTGGACCAAGA-TGTCCTGAG-3′, LAMB1 reverse, 5′-C-AATATATTCTGCCTCCCCG-3′, LAMA3 forward, 5′-GTGGTTACCTCACT-TACCAAGCCA-3′, LAMA3 reverse, 5′-GGTGAGCCTTTGAGTCTCTGT-GAA-3′, LAMB3 forward, 5′-CCAATATCATGCCCTGGTGAGCTA-3′, LAMB3 reverse, 5′-TGCAGAA-CAGTAGCTGAGTCTGTG-3′, CK15 forward, 5′-TGACCTGGAGGTGAAG-ATCC-3′ and CK15 reverse, 5′-GATGGTGGT-GGCCATGAT-3′.

### Colony formation assay

3T3 fibroblasts were cultured in DMEM containing 10% FBS, and subcultured with 1:4 split for several passages when they reached 80–90% confluence. The fibroblasts were killed by exposure to 5 µg/ml mitomycin C for 2 h, and then trypsinized and plated at 5 × 10^5^ cells/well in 6-well culture plates as a feeder layer for colony formation assay. Human normal epidermal keratinocytes obtained from donors of various ages were pre-cultured in Humedia-KG2 (KURABO, Osaka, Japan) in laminin-511-coated or non-coated flasks. After the cells had reached 80% confluence, 5 × 10^2^ keratinocytes/well were seeded into 3T3 fibroblast-cultured 6-well plates^57^. Clonal cultures were maintained in Humedia-KG2 medium for 12 days, fixed with 4% paraformaldehyde and stained with 1% crystal violet.

### Statistical analysis

The data are presented as mean values ± SD. Statistical significance was determined by analysis of variance (ANOVA) and *P* values were calculated using Fisher’s protected least significant difference (Fisher’s PLSD) test.

## Supplementary information

Supplementary information.

## Abbreviations

TEWL: Transepidermal water loss
MMP(s): Matrix metalloproteinase(s)
UVB: Ultraviolet B
HS: Heparan sulfate
MCSP: Melanoma-associated chondroitin sulphate proteoglycan
DEJ: Dermal–epidermal junction
IFE-SCs: Interfollicular epidermal stem cells
BM: Basement membrane
SE: Skin equivalent

## References

[1] Ghadially R (2012). 25 years of epidermal stem cell research. J. Invest. Dermatol..

[2] Cotsarelis G (2006). Epithelial stem cells: A folliculocentric view. J. Invest. Dermatol..

[3] Solanas G, Benitah SA (2013). Regenerating the skin: A task for the heterogeneous stem cell pool and surrounding niche. Nat. Rev. Mol. Cell Biol..

[4] Jones PH, Watt FM (1993). Separation of human epidermal stem cells from transit amplifying cells on the basis of differences in integrin function and expression. Cell.

[5] Jones PH, Harper S, Watt FM (1995). Stem cell patterning and fate in human epidermis. Cell.

[6] Li A, Simmons PJ, Kaur P (1998). Identification and isolation of candidate human keratinocyte stem cells based on cell surface phenotype. Proc. Natl. Acad. Sci. USA.

[7] Lowell S, Jones P, Le Roux I, Dunne J, Watt FM (2000). Stimulation of human epidermal differentiation by delta-notch signalling at the boundaries of stem-cell clusters. Curr. Biol..

[8] Legg J, Jensen UB, Broad S, Leigh I, Watt FM (2003). Role of melanoma chondroitin sulphate proteoglycan in patterning stem cells in human interfollicular epidermis. Development (Cambridge, England).

[9] Jensen KB, Watt FM (2006). Single-cell expression profiling of human epidermal stem and transit-amplifying cells: Lrig1 is a regulator of stem cell quiescence. Proc. Natl. Acad. Sci. USA.

[10] Truzzi F (2015). CD271 mediates stem cells to early progeny transition in human epidermis. J. Invest. Dermatol..

[11] Webb A, Li A, Kaur P (2004). Location and phenotype of human adult keratinocyte stem cells of the skin. Differe. Res. Biol. Divers..

[12] Giangreco A, Goldie SJ, Failla V, Saintigny G, Watt FM (2010). Human skin aging is associated with reduced expression of the stem cell markers beta1 integrin and MCSP. J. Invest. Dermatol..

[13] Akamatsu H (2016). Age-related decrease in CD271(+) cells in human skin. J. Dermatol..

[14] Spradling A, Drummond-Barbosa D, Kai T (2001). Stem cells find their niche. Nature.

[15] Morgner J (2015). Integrin-linked kinase regulates the niche of quiescent epidermal stem cells. Nat. Commun..

[16] Yurchenco PD, Patton BL (2009). Developmental and pathogenic mechanisms of basement membrane assembly. Curr. Pharm. Des..

[17] Lavker RM (1979). Structural alterations in exposed and unexposed aged skin. J. Invest. Dermatol..

[18] Amano S (2009). Possible involvement of basement membrane damage in skin photoaging. J. Investig. Dermatol..

[19] Iriyama S (2011). Activation of heparanase by ultraviolet B irradiation leads to functional loss of basement membrane at the dermal–epidermal junction in human skin. Arch. Dermatol. Res..

[20] Yamada T (2018). Laminin-332 regulates differentiation of human interfollicular epidermal stem cells. Mech. Ageing Dev..

[21] Pouliot N, Saunders NA, Kaur P (2002). Laminin 10/11: an alternative adhesive ligand for epidermal keratinocytes with a functional role in promoting proliferation and migration. Exp. Dermatol..

[22] Kazanis I (2010). Quiescence and activation of stem and precursor cell populations in the subependymal zone of the mammalian brain are associated with distinct cellular and extracellular matrix signals. J. Neurosci..

[23] Nascimento MA, Sorokin L, Coelho-Sampaio T (2018). Fractone bulbs derive from ependymal cells and their laminin composition influence the stem cell niche in the subventricular zone. J. Neurosci..

[24] Romagnoli M (2019). Deciphering the mammary stem cell niche: A role for laminin-binding integrins. Stem Cell Rep..

[25] Yao Y, Norris EH, Mason CE, Strickland S (2016). Laminin regulates PDGFRbeta(+) cell stemness and muscle development. Nat. Commun..

[26] Rayagiri SS (2018). Basal lamina remodeling at the skeletal muscle stem cell niche mediates stem cell self-renewal. Nat. Commun..

[27] Amano S (2005). Protective effect of matrix metalloproteinase inhibitors against epidermal basement membrane damage: Skin equivalents partially mimic photoageing process. Br. J. Dermatol..

[28] Iriyama S, Hiruma T, Tsunenaga M, Amano S (2011). Influence of heparan sulfate chains in proteoglycan at the dermal–epidermal junction on epidermal homeostasis. Exp. Dermatol..

[29] Iriyama S, Matsuura-Hachiya Y, Tsunenaga M (2018). Influence of epidermal basement membrane integrity on cutaneous permeability barrier function. J. Dermatol. Sci..

[30] Iriyama S, Yamanishi H, Kunizawa N, Hirao T, Amano S (2019). 1-(2-Hydroxyethyl)-2-imidazolidinone, a heparanase and matrix metalloproteinase inhibitor, improves epidermal basement membrane structure and epidermal barrier function. Exp. Dermatol..

[31] Feige P, Brun CE, Ritso M, Rudnicki MA (2018). Orienting muscle stem cells for regeneration in homeostasis, aging, and disease. Cell Stem Cell.

[32] Lopez-Otin C, Blasco MA, Partridge L, Serrano M, Kroemer G (2013). The hallmarks of aging. Cell.

[33] Molofsky AV (2006). Increasing p16INK4a expression decreases forebrain progenitors and neurogenesis during ageing. Nature.

[34] Gruber R (2006). Fracture healing in the elderly patient. Exp. Gerontol..

[35] Conboy IM, Rando TA (2012). Heterochronic parabiosis for the study of the effects of aging on stem cells and their niches. Cell Cycle.

[36] Liu Y, Lyle S, Yang Z, Cotsarelis G (2003). Keratin 15 promoter targets putative epithelial stem cells in the hair follicle bulge. J. Invest. Dermatol..

[37] Liao YH (2014). Quantitative analysis of intrinsic skin aging in dermal papillae by in vivo harmonic generation microscopy. Biomed. Opt. Express.

[38] Mizukoshi K (2015). Changes in dermal papilla structures due to aging in the facial cheek region. Skin Res. Technol..

[39] Haytoglu NS (2014). Assessment of skin photoaging with reflectance confocal microscopy. Skin Res. Technol..

[40] Abboud-Jarrous G (2008). Cathepsin L is responsible for processing and activation of proheparanase through multiple cleavages of a linker segment. J. Biol. Chem..

[41] Miyazaki T (2012). Laminin E8 fragments support efficient adhesion and expansion of dissociated human pluripotent stem cells. Nat. Commun..

[42] Nakagawa M (2014). A novel efficient feeder-free culture system for the derivation of human induced pluripotent stem cells. Sci. Rep..

[43] Tjin MS (2018). Biologically relevant laminin as chemically defined and fully human platform for human epidermal keratinocyte culture. Nat. Commun..

[44] Kikkawa Y, Sanzen N, Fujiwara H, Sonnenberg A, Sekiguchi K (2000). Integrin binding specificity of laminin-10/11: Laminin-10/11 are recognized by alpha 3 beta 1, alpha 6 beta 1 and alpha 6 beta 4 integrins. J. Cell Sci..

[45] Lechler T, Fuchs E (2005). Asymmetric cell divisions promote stratification and differentiation of mammalian skin. Nature.

[46] Klaffky EJ, Gonzales IM, Sutherland AE (2006). Trophoblast cells exhibit differential responses to laminin isoforms. Dev. Biol..

[47] Adair-Kirk TL (2012). Keratinocyte-targeted expression of human laminin gamma2 rescues skin blistering and early lethality of laminin gamma2 deficient mice. PLoS ONE.

[48] Schluter H, Stark HJ, Sinha D, Boukamp P, Kaur P (2013). WIF1 is expressed by stem cells of the human interfollicular epidermis and acts to suppress keratinocyte proliferation. J. Invest. Dermatol..

[49] Hohenester E, Yurchenco PD (2013). Laminins in basement membrane assembly. Cell Adhes. Migr..

[50] Lin L, Kurpakus-Wheater M (2002). Laminin alpha5 chain adhesion and signaling in conjunctival epithelial cells. Invest. Ophthalmol. Vis. Sci..

[51] Kammerer RA (1999). Interaction of agrin with laminin requires a coiled-coil conformation of the agrin-binding site within the laminin gamma1 chain. EMBO J..

[52] Iriyama S, Yamanihi H, Kunizawa N, Hirao T, Amano S (2019). 1-(2-Hydroxyethyl)-2-imidazolidinone, a heparanase and matrix metalloproteinase inhibitor, improves epidermal basement membrane structure and epidermal barrier function. Exp. Dermatol..

[53] Liu N (2019). Stem cell competition orchestrates skin homeostasis and ageing. Nature.

[54] Iriyama S, Tsunenaga M, Amano S, Adachi E (2011). Key role of heparan sulfate chains in assembly of anchoring complex at the dermal–epidermal junction. Exp. Dermatol..

[55] Pan W (2006). 1-[4-(1H-Benzoimidazol-2-yl)-phenyl]-3-[4-(1H-benzoimidazol-2-yl)-phenyl]-urea derivatives as small molecule heparanase inhibitors. Bioorg. Med. Chem. Lett..

[56] MacPherson LJ (1997). Discovery of CGS 27023A, a non-peptidic, potent, and orally active stromelysin inhibitor that blocks cartilage degradation in rabbits. J. Med. Chem..

[57] Rheinwald JG, Green H (1975). Serial cultivation of strains of human epidermal keratinocytes: The formation of keratinizing colonies from single cells. Cell.

