# Supplementary material for: Decrease of laminin-511 in the basement membrane due to photoaging reduces epidermal stem/progenitor cells
Source: Sci Rep. 2020 Jul 28;10:12592. doi: 10.1038/s41598-020-69558-y (PMC7387558; doi:10.1038/s41598-020-69558-y)
Supplement: Supplementary file 1 — Supplementary information. [file 41598_2020_69558_MOESM1_ESM.docx]

**Supplementary Information**

**Title:** Decrease of laminin-511 in the basement membrane due to photoaging reduces epidermal stem/progenitor cells

**Authors:** Shunsuke Iriyama, Masahito Yasuda, Saori Nishikawa, Eisuke Takai, Junichi Hosoi and Satoshi Amano

**
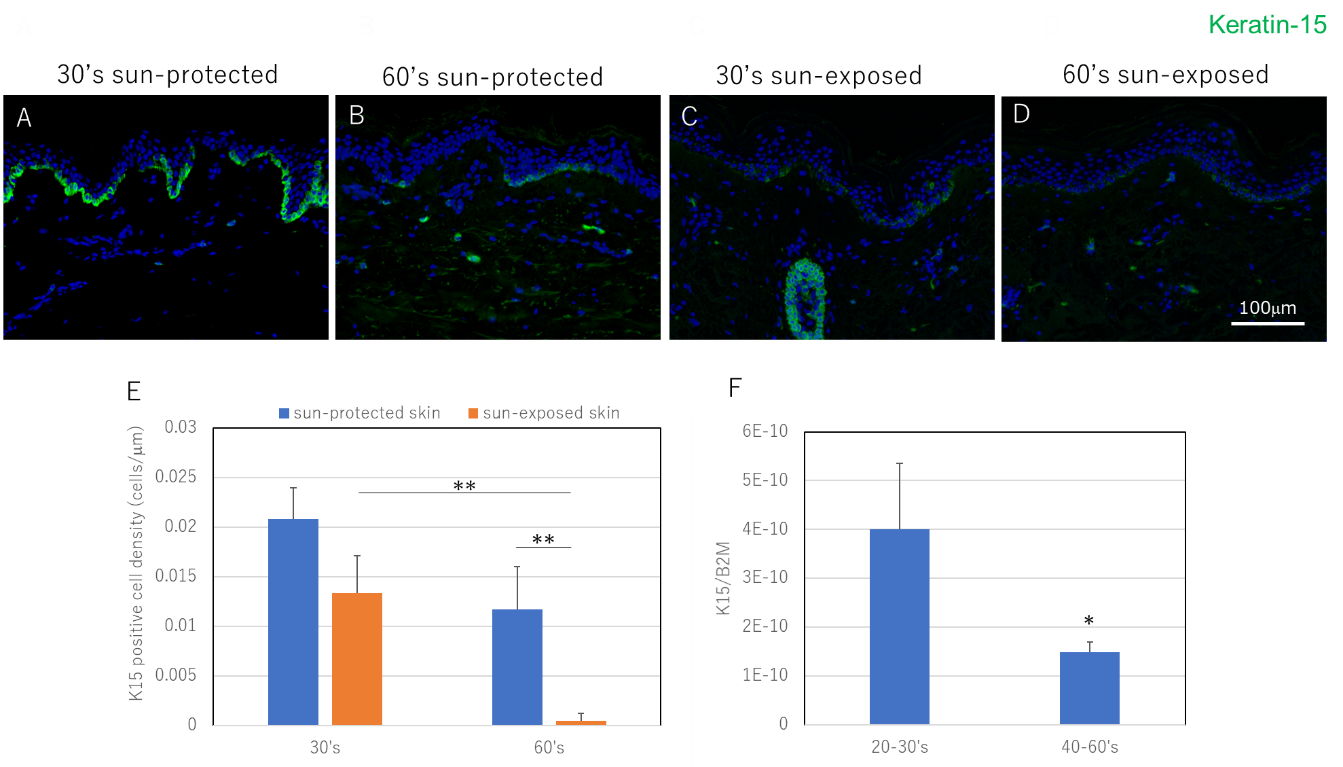
**

**Supplementary Figure S1 Age-dependent change of cytokeratin-15-positive cells in sun-protected skin and sun-exposed skin**

Immunofluorescence staining of cytokeratin-15 (green) in 30’s sun-protected skin (A), 60’s sun-protected skin (B), 30’s sun-exposed skin (C) and 60’s sun-exposed skin (D). K15-positive cell density was analyzed using WINROOF 2013 image analyzing software (Mitani, Fukui, Japan, https://www.mitani- visual.jp/products/image_analys_ismeasure-ment/winroof/) (E). mRNA expression levels of KRT15 were analyzed by qPCR (E). Data are expressed as the mean ± SD from each of the 6 donors in Fig.S1E, and from each of the 5 donors in Fig.S1F. Bars: 100 μm. *;p<0.05, **; p < 0.01.


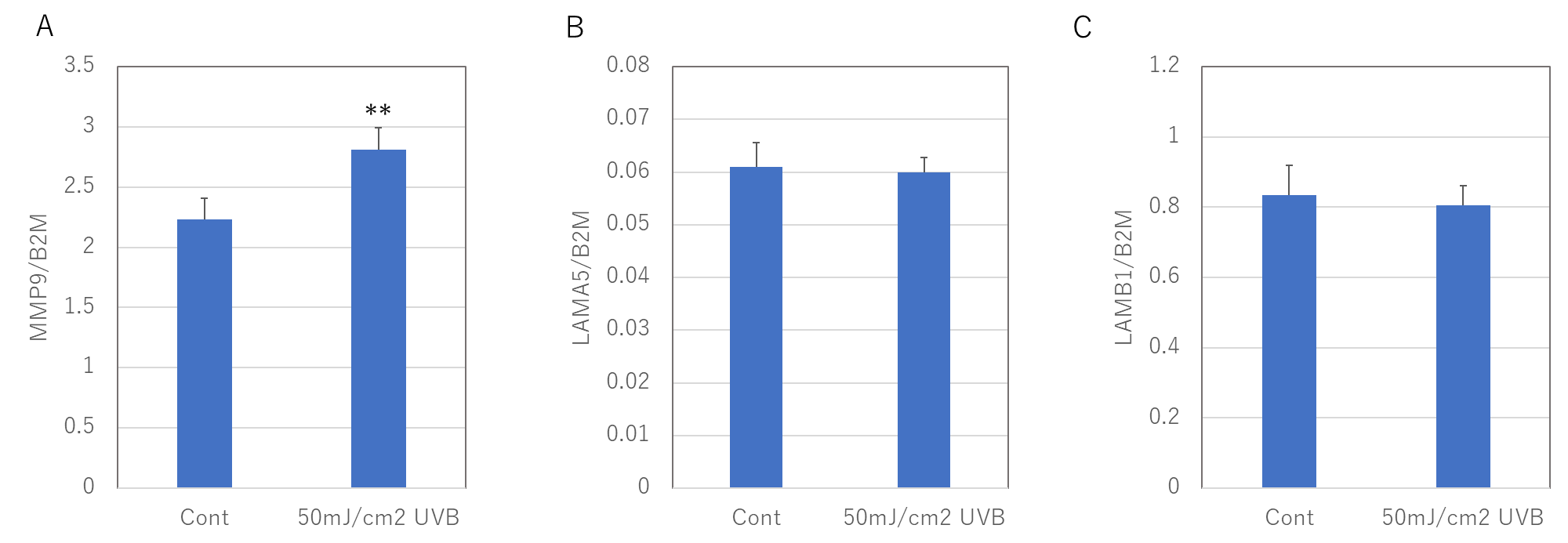


**Supplementary Figure S2　Effect of 50 mJ/cm^2^ UVB irradiation on gene expression levels of MMP9, LAMA5 and LAMB1 in cultured keratinocytes**

mRNA expression levels of MMP9 (A), LAMA5 (B) and LAMB1 (C) were analyzed by qPCR. Data are expressed as mean ± SD. **; p < 0.01.


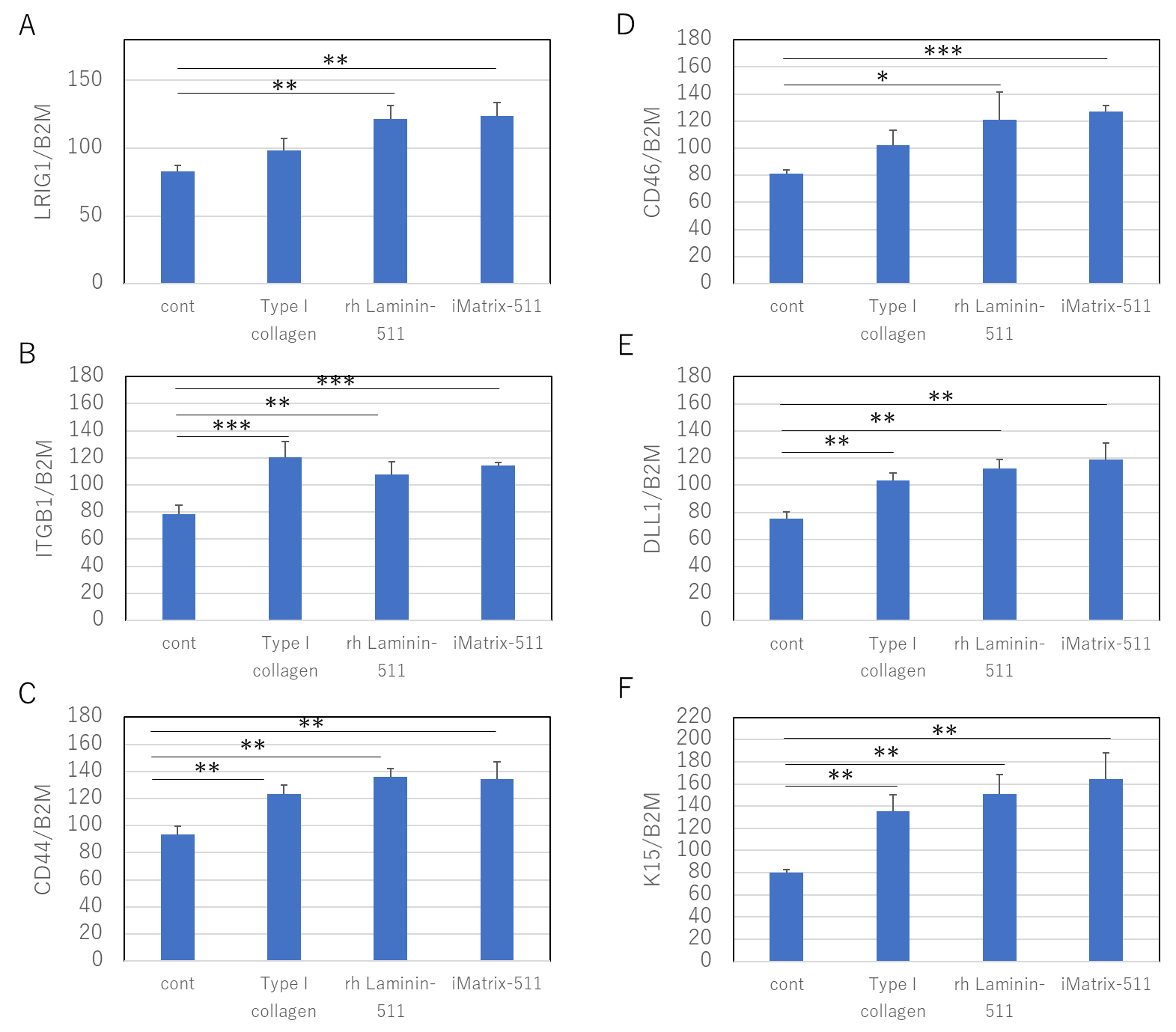


**Supplementary Figure S3 Epidermal stem cell markers were increased in the presence of laminin-511**

The mRNA expression levels of LRIG1 (A), ITGB1 (B), CD44 (C), CD46 (D), DLL1 (E), and K15 (F) were analyzed by qPCR. Data are expressed as the mean ± SD from the 3 donors. Statistical analysis was performed using ANOVA with the Tukey–Kramer post hoc test: *;p<0.05, **; p<0.01, ***;p<0.01. Bars: 100 μm.

**
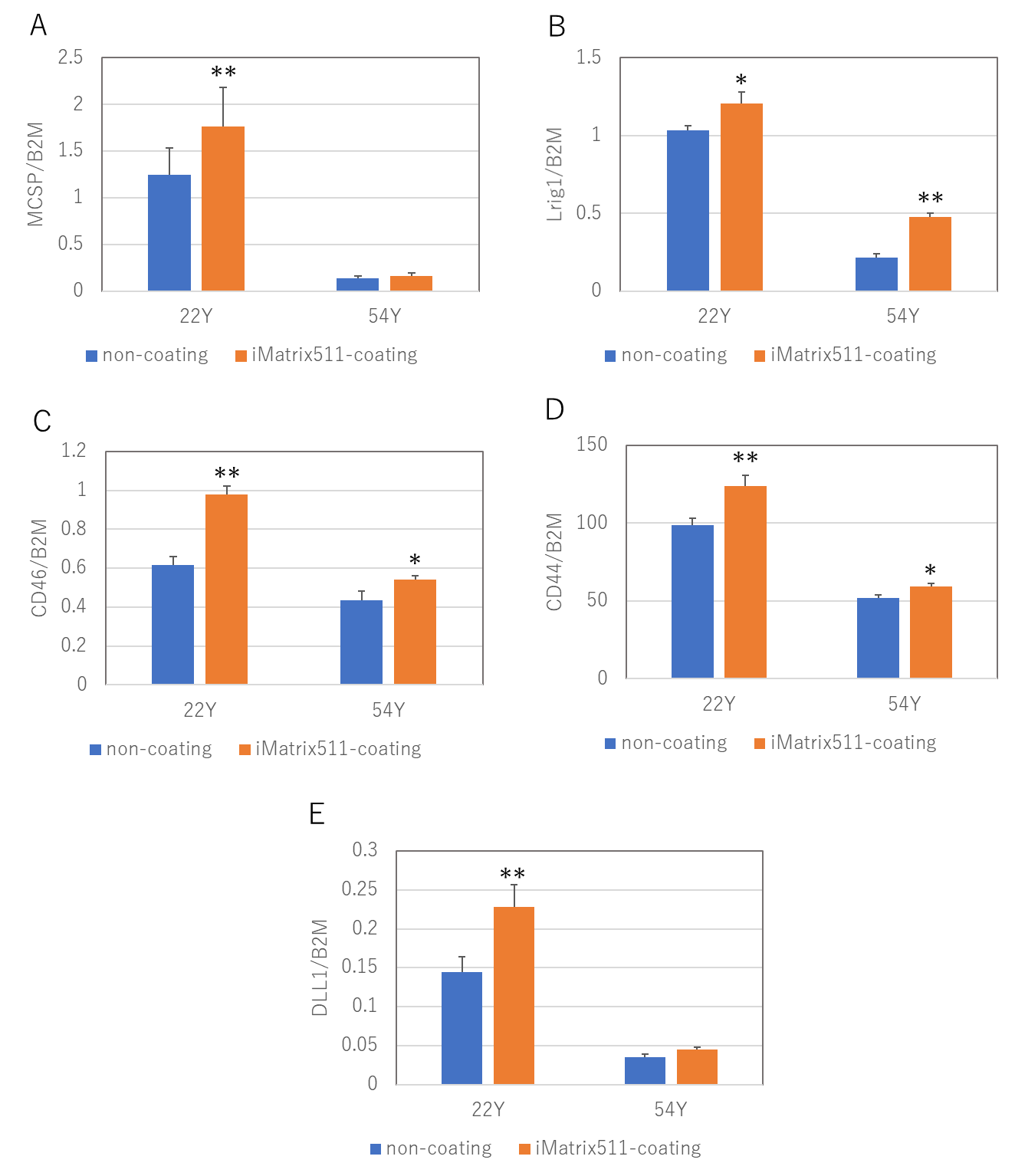
**

**Supplementary Figure S4 Epidermal stem cell markers were increased in the presence of iMatrix-511**

The mRNA expression levels of MCSP (A), Lrig1 (B), CD46 (C), CD44 (D) and DLL1 (E) in cultured adult keratinocytes from 22-year-old and 54-year-old subjects were analyzed by qPCR. Data are expressed as mean ± SD. *; p < 0.05, **; p<0.01.


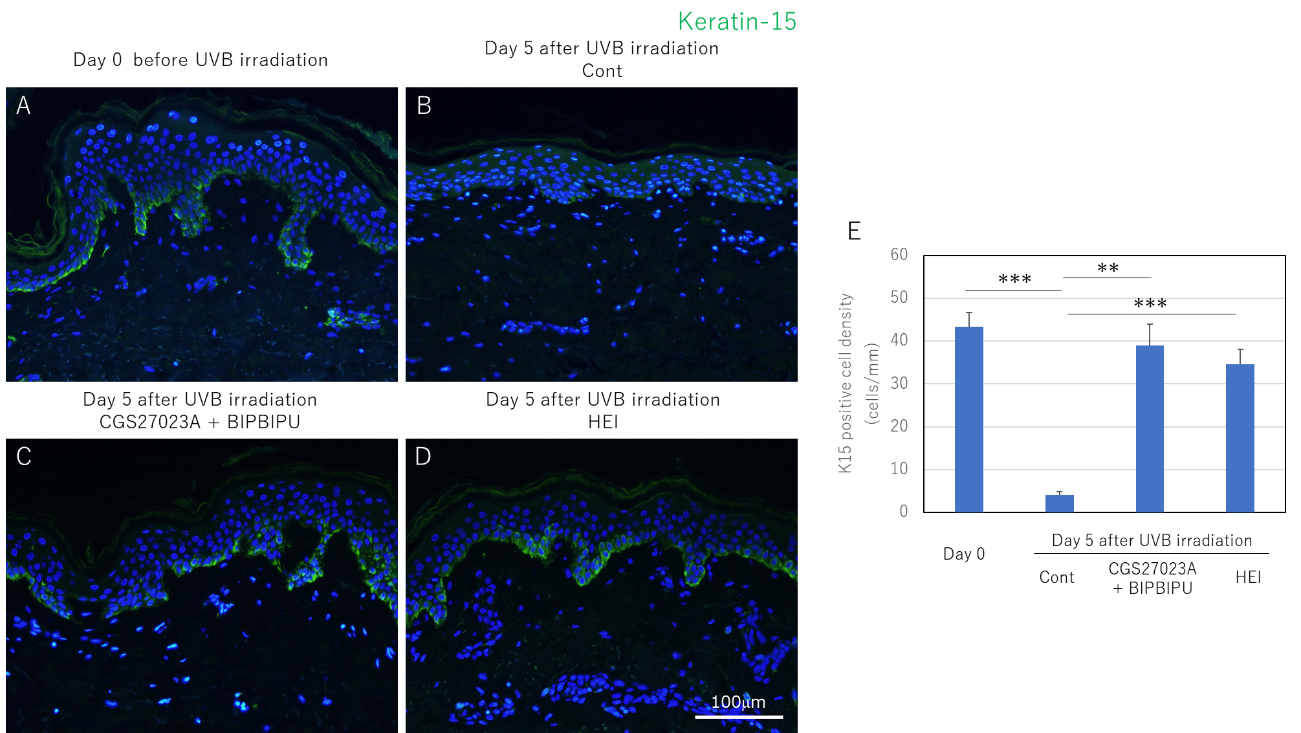


**Supplementary Figure S5 Epidermal stem/progenitor cells increased in the presence of the MMP inhibitor and the heparanase inhibitor in the UVB-exposed OC model**

Immunofluorescence staining intensities of keratin-15 (A-D) were compared in un-cultured skin (A), and in UVB-exposed organotypic human skin without treatment (B), after treatment with MMP inhibitor CGS27023A and heparanase inhibitor BIPBIPU (C), and after treatment with bifunctional inhibitor HEI (D). K15-positive cell density was analyzed using WINROOF2013 image analyzing software (Mitani, Fukui, Japan, <https://www.mitani-visual.jp/products/image_analys_ismeasurement/winroof/>)　(E). Data are expressed as mean ± SD from each of the 4 donors in Fig. S6E. **;p<0.01, ***; p < 0.001. Bar, 100 μm.

**Supplementary Table S1 Source of human skin for histological experiments**

**
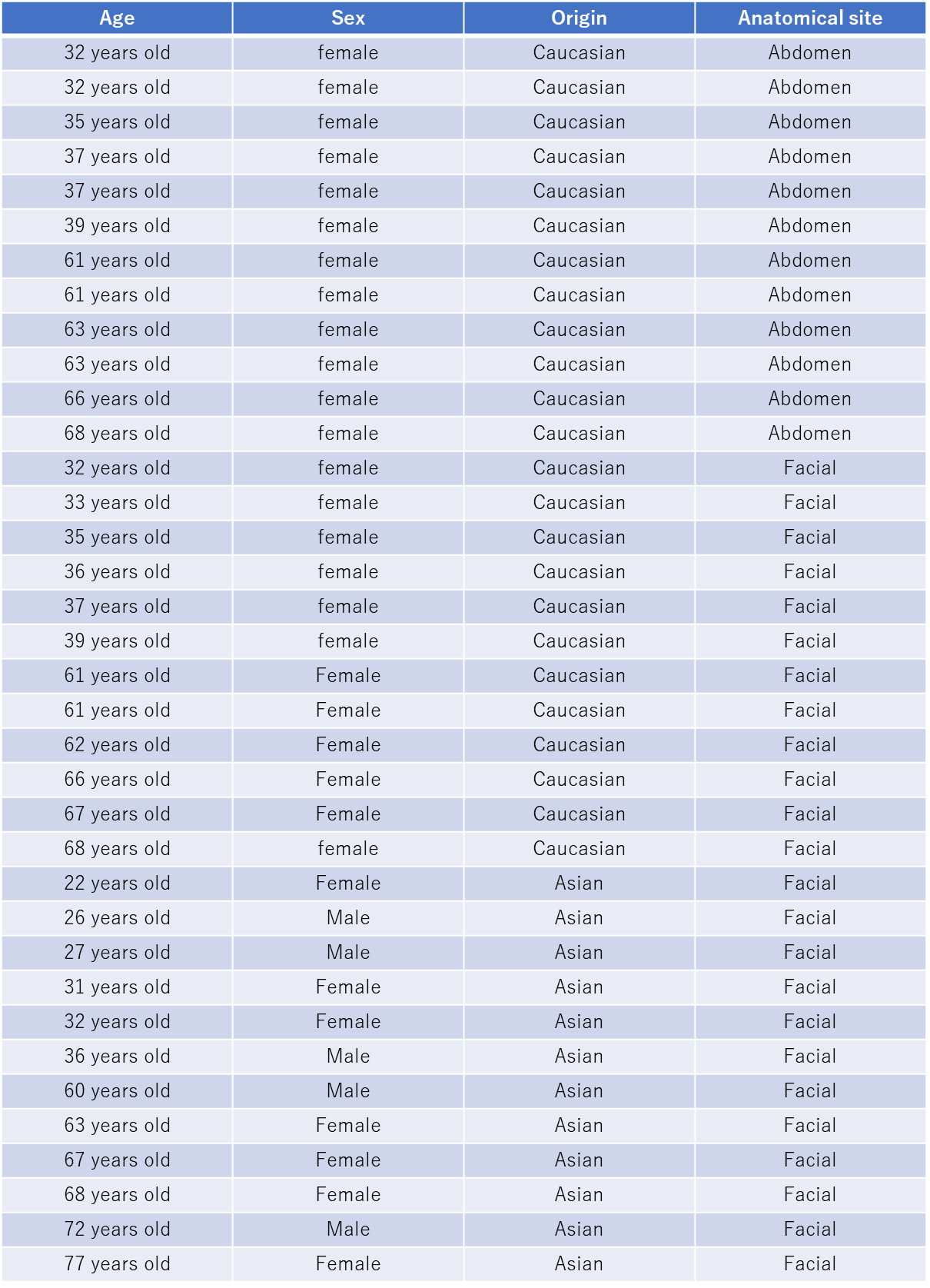
**

**Supplementary Table S2　Source of organotypic human skin**


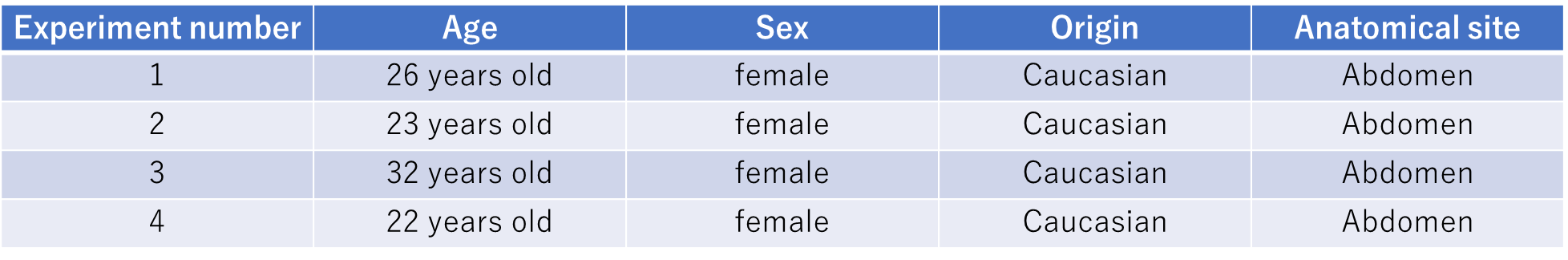


**Supplementary Table S3　Source of cultured keratinocytes**

**
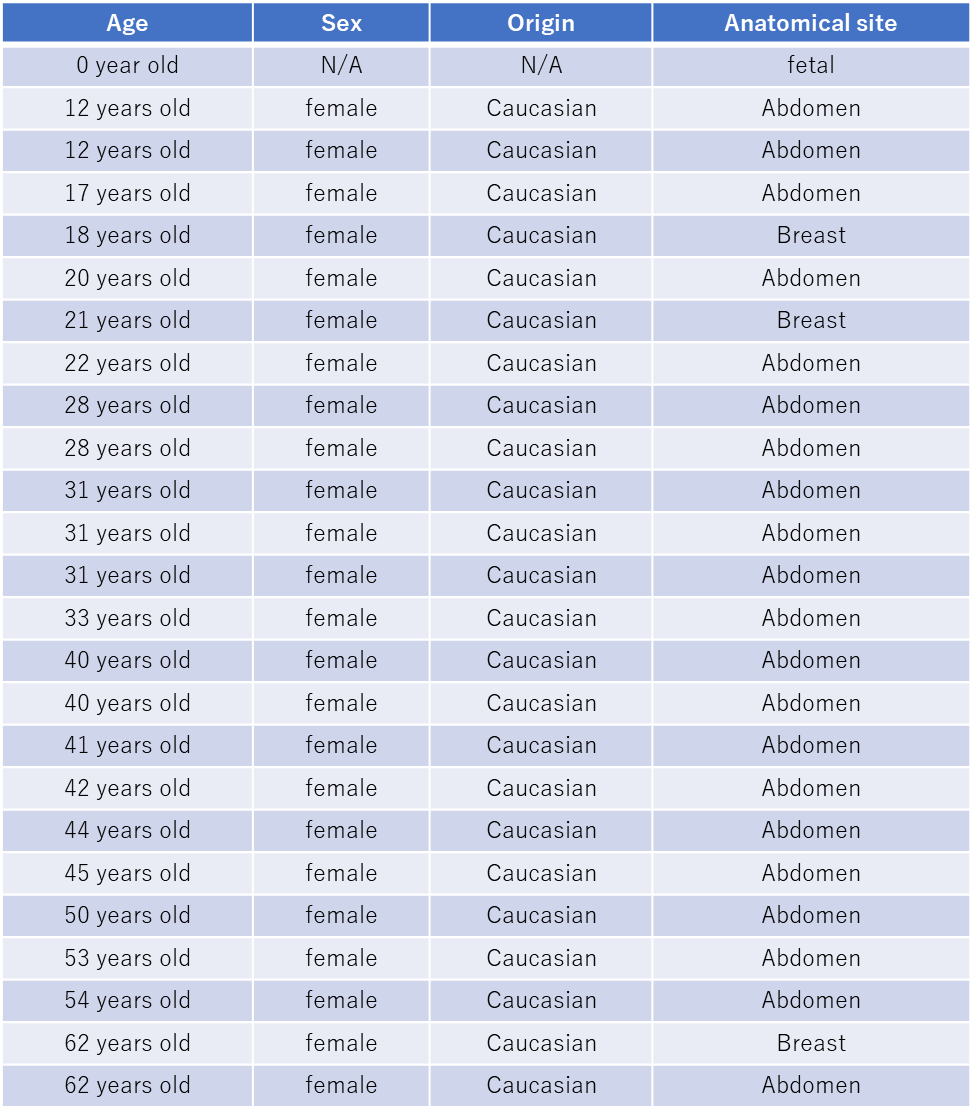
**
